# Supplementary figures and images for: Motor Cortical Neuronal Hyperexcitability Associated with α-Synuclein Aggregation
Source: bioRxiv. 2024 Aug 14:2024.07.24.604995. Originally published 2024 Jul 24. Preprint. [Version 2] doi: 10.1101/2024.07.24.604995 (PMC11291145; doi:10.1101/2024.07.24.604995)

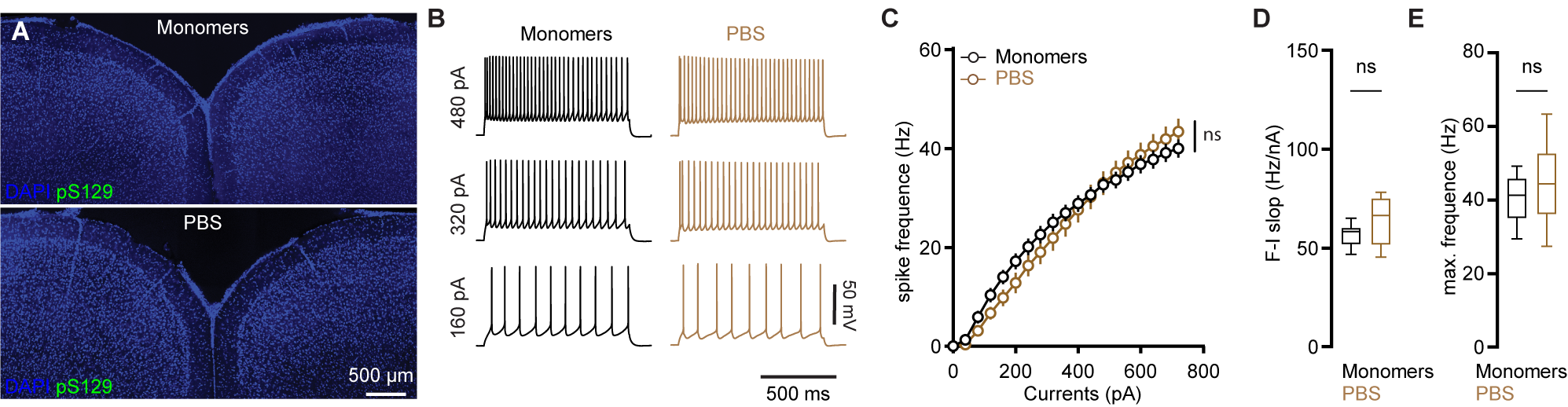

Supplement: Supplement 1 [file media-1.tif]

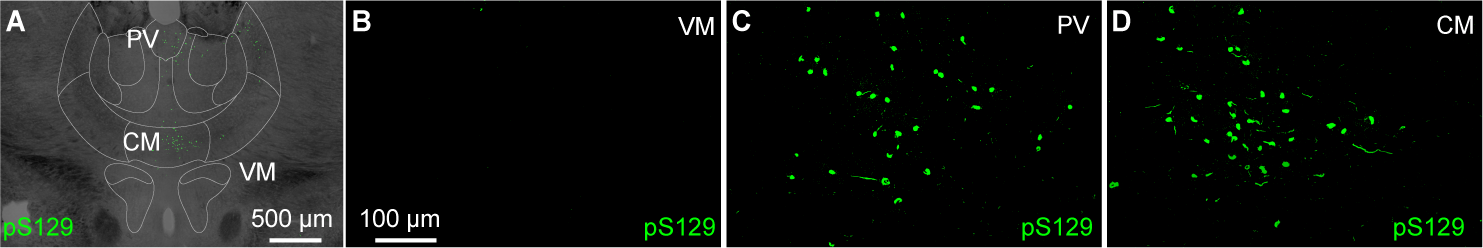

Supplement: Supplement 2 [file media-2.tif]

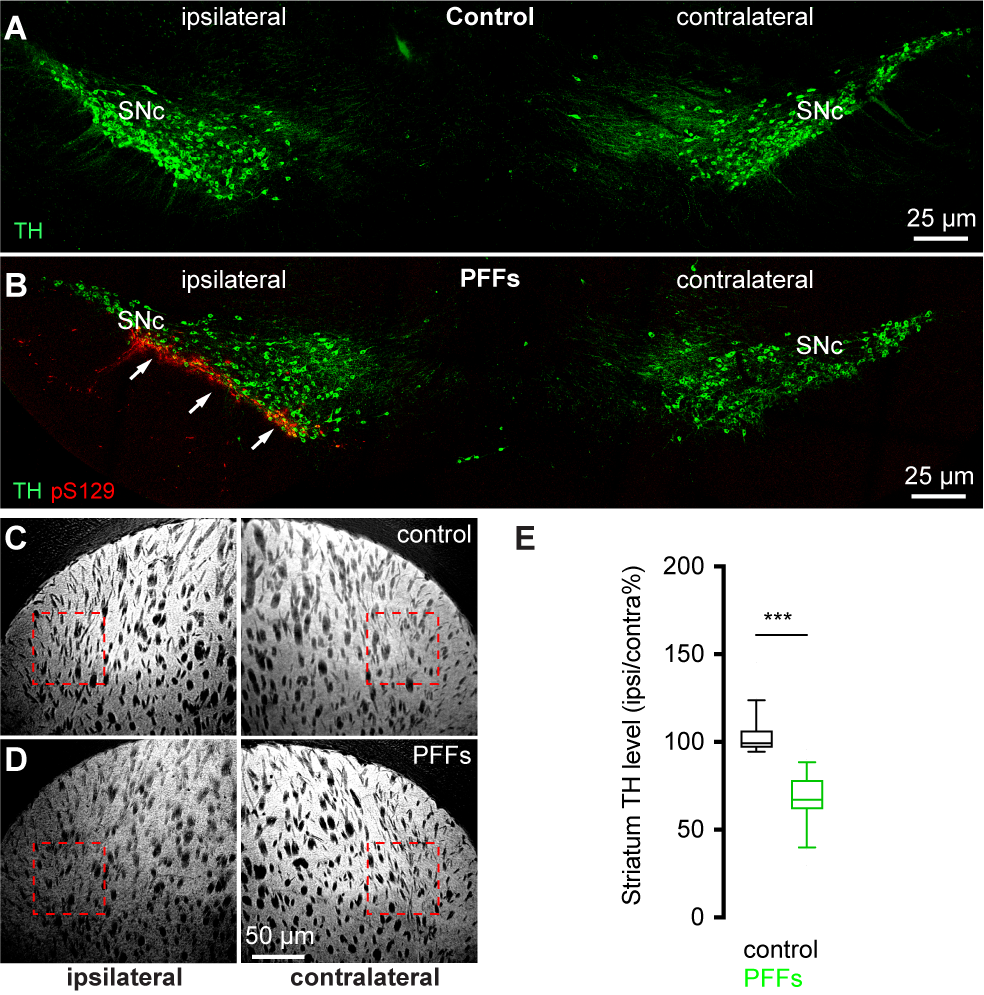

Supplement: Supplement 3 [file media-3.tif]
